# Aberrant growth of the anterior cranial base relevant to severe midface hypoplasia of Apert syndrome

**DOI:** 10.1186/s40902-018-0179-8

**Published:** 2018-12-12

**Authors:** Bong Kuen Cha, Dong Soon Choi, In San Jang, Hyun Tae Yook, Seung Youp Lee, Sang Shin Lee, Suk Keun Lee

**Affiliations:** 1 Department of Orthodontics, College of Dentistry, Gangneung-Wonju National University, Gangneung, South Korea; 2 Department of Orthodontics, College of Dentistry, Chunbuk National University, Jeonju, South Korea; 3 Department of Oral Pathology, College of Dentistry, Gangneung-Wonju National University, 123 Chibyun-dong, Gangneung, 210-702 South Korea

**Keywords:** Apert syndrome, Midface hypoplasia, Anterior cranial base, Retruded zygomatic axes

## Abstract

**Background:**

A 9-year-old male showed severe defects in midface structures, which resulted in maxillary hypoplasia, ocular hypertelorism, relative mandibular prognathism, and syndactyly. He had been diagnosed as having Apert syndrome and received a surgery of frontal calvaria distraction osteotomy to treat the steep forehead at 6 months old, and a surgery of digital separation to treat severe syndactyly of both hands at 6 years old. Nevertheless, he still showed a turribrachycephalic cranial profile with proptosis, a horizontal groove above supraorbital ridge, and a short nose with bulbous tip.

**Methods:**

Fundamental aberrant growth may be associated with the cranial base structure in radiological observation.

**Results:**

The Apert syndrome patient had a shorter and thinner nasal septum in panthomogram, PA view, and Waters’ view; shorter zygomatico-maxillary width (83.5 mm) in Waters’ view; shorter length between the sella and nasion (63.7 mm) on cephalogram; and bigger zygomatic axis angle of the cranial base (118.2°) in basal cranial view than a normal 9-year-old male (94.8 mm, 72.5 mm, 98.1°, respectively). On the other hand, the Apert syndrome patient showed interdigitating calcification of coronal suture similar to that of a normal 30-year-old male in a skull PA view.

**Conclusion:**

Taken together, the Apert syndrome patient, 9 years old, showed retarded growth of the anterior cranial base affecting severe midface hypoplasia, which resulted in a hypoplastic nasal septum axis, retruded zygomatic axes, and retarded growth of the maxilla and palate even after frontal calvaria distraction osteotomy 8 years ago. Therefore, it was suggested that the severe midface hypoplasia and dysostotic facial profile of the present Apert syndrome case are closely relevant to the aberrant growth of the anterior cranial base supporting the whole oro-facial and forebrain development.

## Background

Apert syndrome is a rare autosomal dominant disorder characterized by severe syndactyly of the feet and hands, craniofacial abnormalities, and craniosynostosis, which is also known to be caused by one of the two specific point mutations in the fibroblast growth factor receptor 2 (FGFR2), i.e., Ser252Trp and Pro253Arg [1–3]. There was a trend of more frequent amblyopia and strabismus in FGFR2 Ser252Trp mutation and more frequent optic disc pallor in the FGFR2 Pro253Arg mutation [4, 5]. These differential effects of FGFR2 mutations in ophthalmic findings in patients with Apert syndrome, with significantly greater prevalence of visual impairment in the Ser252Trp mutation compared with that in the Pro253Arg mutation, may imply a developmental etiology of mutated FGFR2 proteins which can function differently [6]. The abnormal overexpression or accumulation of proteoglycan, i.e., decorin and biglycan, was found and presumed to be caused by the low affinity of receptor regulation in FGFR2 signaling cascade, which resulted in premature periosteal ossification inducing membranous bone dysostosis [7].

When normal development and growth of the calvarial sutures is disrupted, craniosynostosis leading to an abnormal head shape, ocular hypertelorism with proptosis, and midface hypoplasia may result. Classical craniosynostosis syndromes are autosomal dominant traits and include Apert, Pfeiffer, Crouzon, Jackson-Weiss, and Saethre-Chotzen syndromes [8]. Apert syndrome is the most common among acrocephalosyndactylies with complex malformations of the hands. The Apert hand requires early and specialized treatment that aims to provide a functional hand before 2 or 3 years, with the least surgical complications. But the functional prognosis is darkened by symphalangism [9, 10].

The craniosynostosis of those with Apert syndrome is usually associated with midface hypoplasia, exhibiting retruded maxilla, undergrowth of the nasal organ, septo-optic dysplasia, and other systemic malformations including mental retardation [11]. Patients with Apert syndrome frequently showed progressive widening of the skull base even after cranioplasty for bilateral coronal craniosynostosis [12].

Although the craniofacial dysostosis of those with Apert syndrome may give great impact on the whole craniofacial growth, the craniofacial structures of those with Apert syndrome have not been precisely analyzed so far due to their complicated and heterogeneous developmental components. The present study tried to analyze the radiological measurements of craniofacial structures obtained from a 9-year-old Apert syndrome patient in comparison with those of an age-matched normal male.

## Case report

This case report has been approved by the Institutional Review Board (IRB2016–11). In order to explore his cranial base structures, the radiograms of panthomogram, posterior to anterior (PA) view, Waters’ view, cephalogram, and basal cranial view were taken and compared with those of the control (age-matched normal male subject). And the dental plaster model was also made to illustrate the precise situation of dental dimensions and palatal shape. Every radiological measurement performed in the methods previously described was analyzed for the significant abnormalities of Apert syndrome compared to the control [13].

**Fig. 1:**
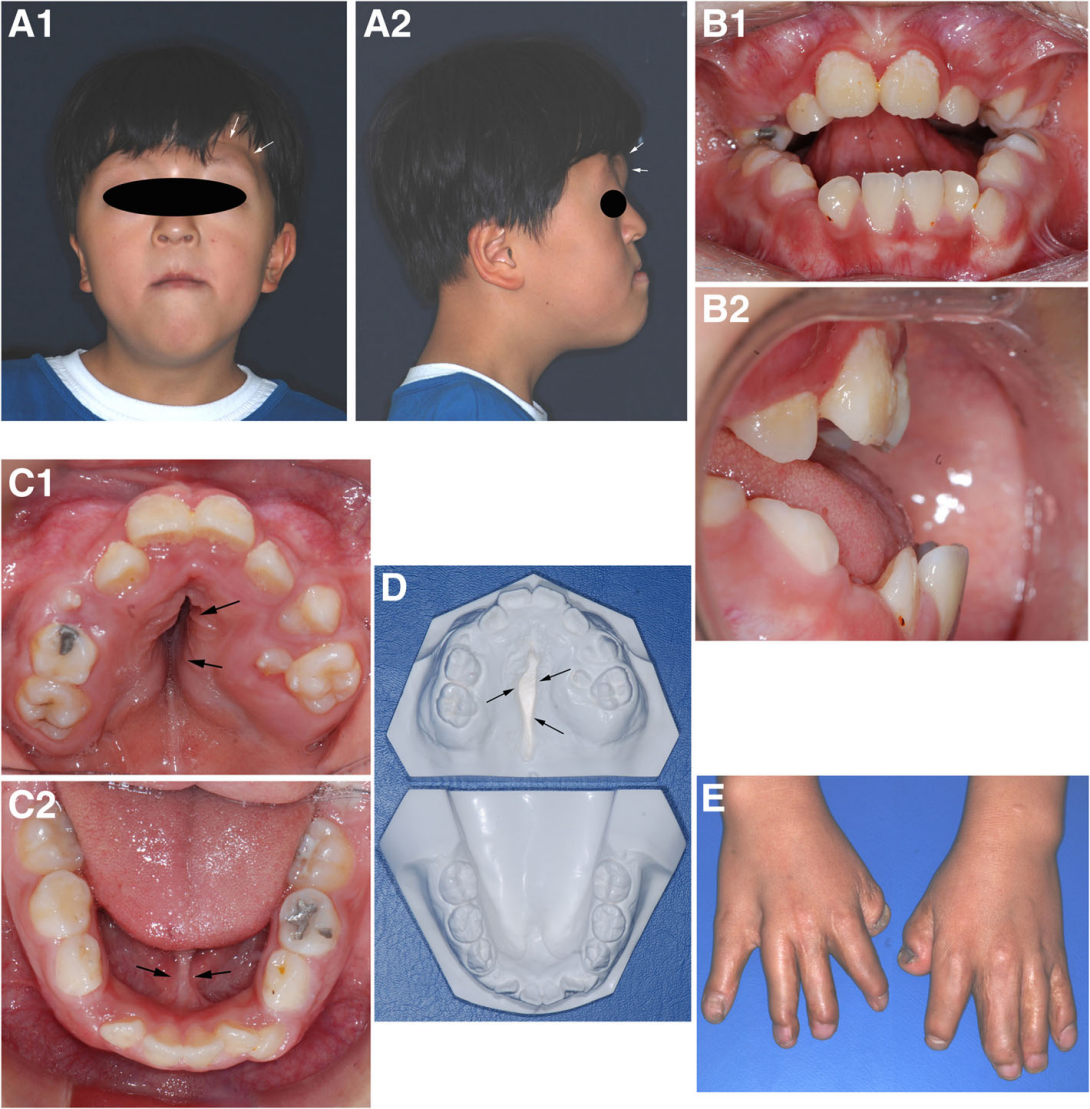
Photograph of an Apert syndrome patient, a 9-year-old male. Facial profile: frontal view (**A1**): hypertelorism, turribrachycephalic cranial profile, horizontal groove above supra orbital ridge (arrows), and a short nose with bulbous tip. Lateral view (**A2**): retarded maxilla and frontal proptosis (arrows). Intraoral view: anterior view (**B1**): anterior open bite and poor oral hygiene. Lateral view (**B2**): retruded maxillary incisors. Upper occlusal view (**C1**): narrow and triangular shape maxillary arch with Byzantine arch-shaped palate. Lower occlusal view (**C2**): almost normal U-shaped mandibular arch with thick lingual frenum (arrows). **D** Plaster dental model: fissure-like palatal groove (arrows). **E** Hand, incompletely separated and with short fingers even after syndactyly operation performed 3 years ago

A 9-year-old male, diagnosed as Apert syndrome after birth, was examined for his oro-facial abnormalities. Besides the severe syndactyly and cardiac anomaly of patent ductal arteriosus, he showed characteristic syndromic features of craniofacial synostosis, i.e., steep forehead, hypertelorism, turribrachycephalic cranial profile, horizontal groove above supra orbital ridge, and short nose with bulbous tip in anterior facial profile, retruded maxilla and frontal proptosis in lateral facial profile, maxillary hypoplasia, and relative mandibular prognathism, (Fig. 1a). Intraoral observation showed severe anterior open bite with poor oral hygiene. His maxillary incisors were severely retruded compared to mandibular incisors, which resulted in an anterior open bite (Fig. 1b). Upper dental arch was much narrow and formed triangular shape palate similar to a Byzantine arch, while mandibular arch was almost normal in U-shape attached with thick lingual frenum (Fig. 1c). Mid-palatal area was deeply grooved in a fissure-like fashion, but nasal perforation was not found (Fig. 1d). His hand still showed short fingers incompletely separated even after syndactyly operation was performed 3 years ago (Fig. 1e).

**Fig. 2:**
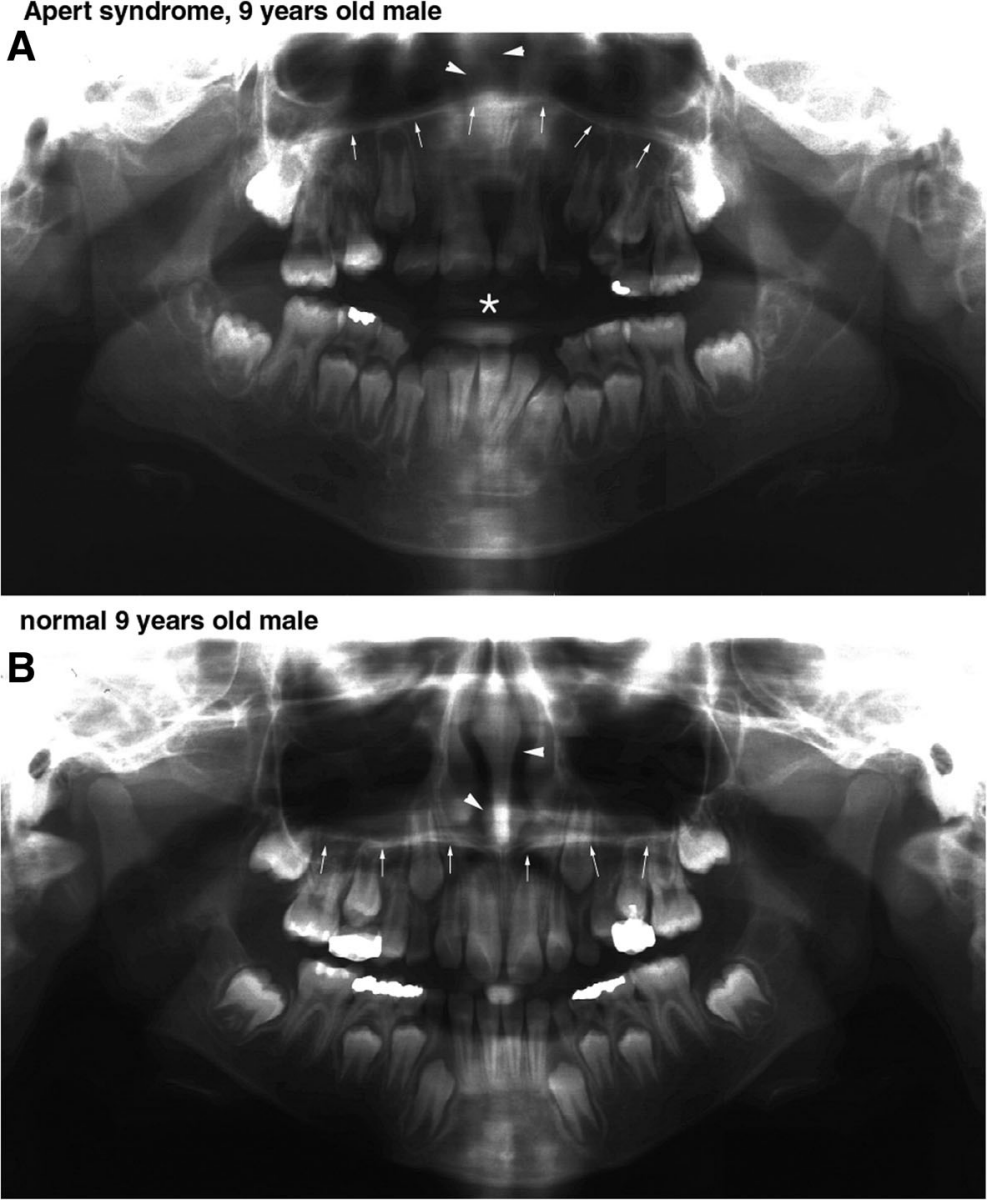
Panoramic view. **a** An Apert syndrome patient, a 9-year-old male, with an upward convex palatal plate (arrows) and a short and thin nasal septum (arrow heads). Noted the severe anterior open bite (*). **b** A 9-year-old normal male with normal horizontal palatal plate (arrows) and a long and thick nasal septum (arrow heads)

In panthomogram, the Apert syndrome patient showed curved palatal plate which was upwardly convex and a relatively short and thin nasal septum, while the control showed horizontal palatal plate and a long and thick nasal septum (Fig. 2).

**Fig. 3:**
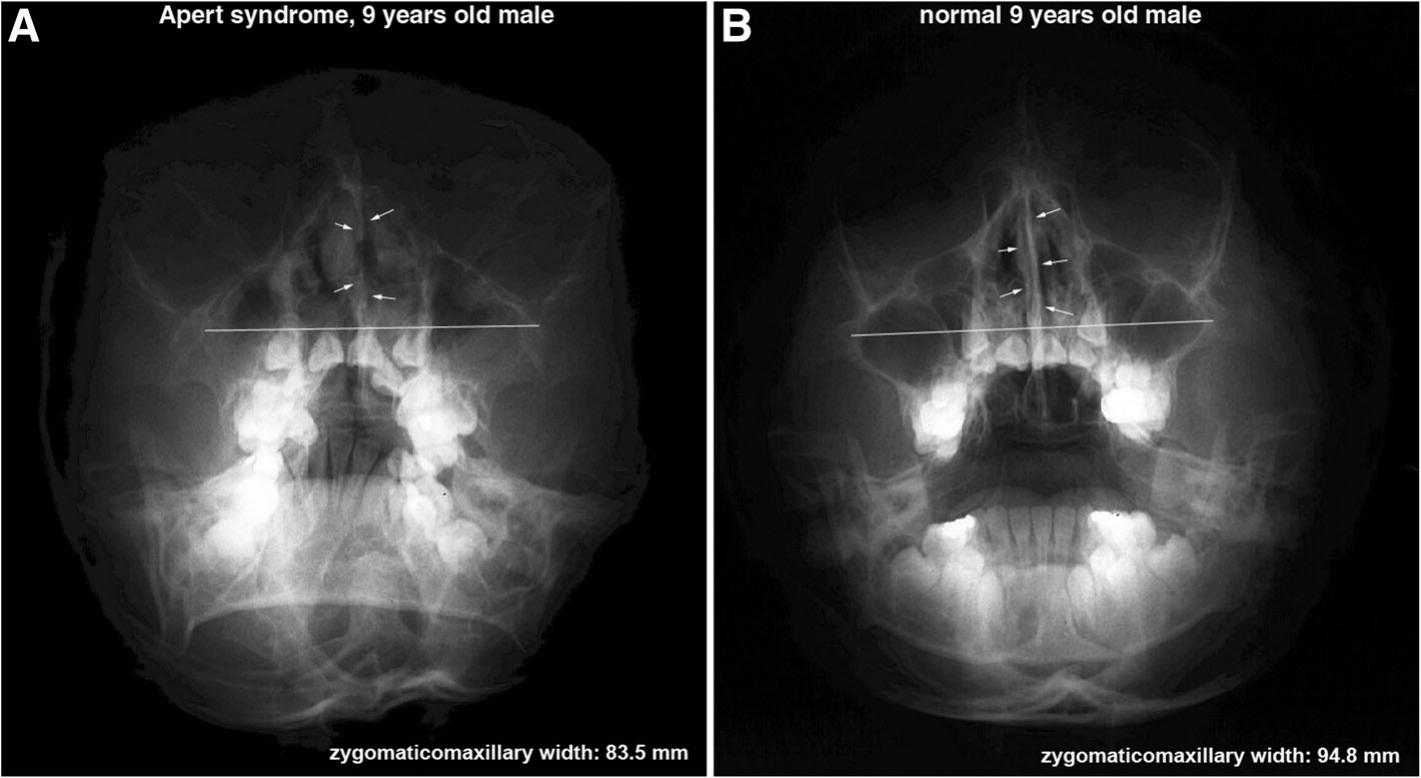
Water’s view: An Apert syndrome patient (**a**), a 9-year-old male, showed a shorter zygomatico-maxillary width and a shorter and thinner nasal septum than a 9-year-old normal male (**b**)

In Waters’ view, the length between the bilateral condensed buccal bony areas produced by the attachment of maxillary process of zygomatic bone to the lateral process of the maxilla may represent the width of maxillary proper and has an implication for the growth of the maxilla; therefore, it was named zygomatico-maxillary width in this study. However, the zygomatico-maxillary width was much reduced in the Apert syndrome patient (83.5 mm) than in the control (94.8 mm) (Fig. 3).

**Fig. 4:**
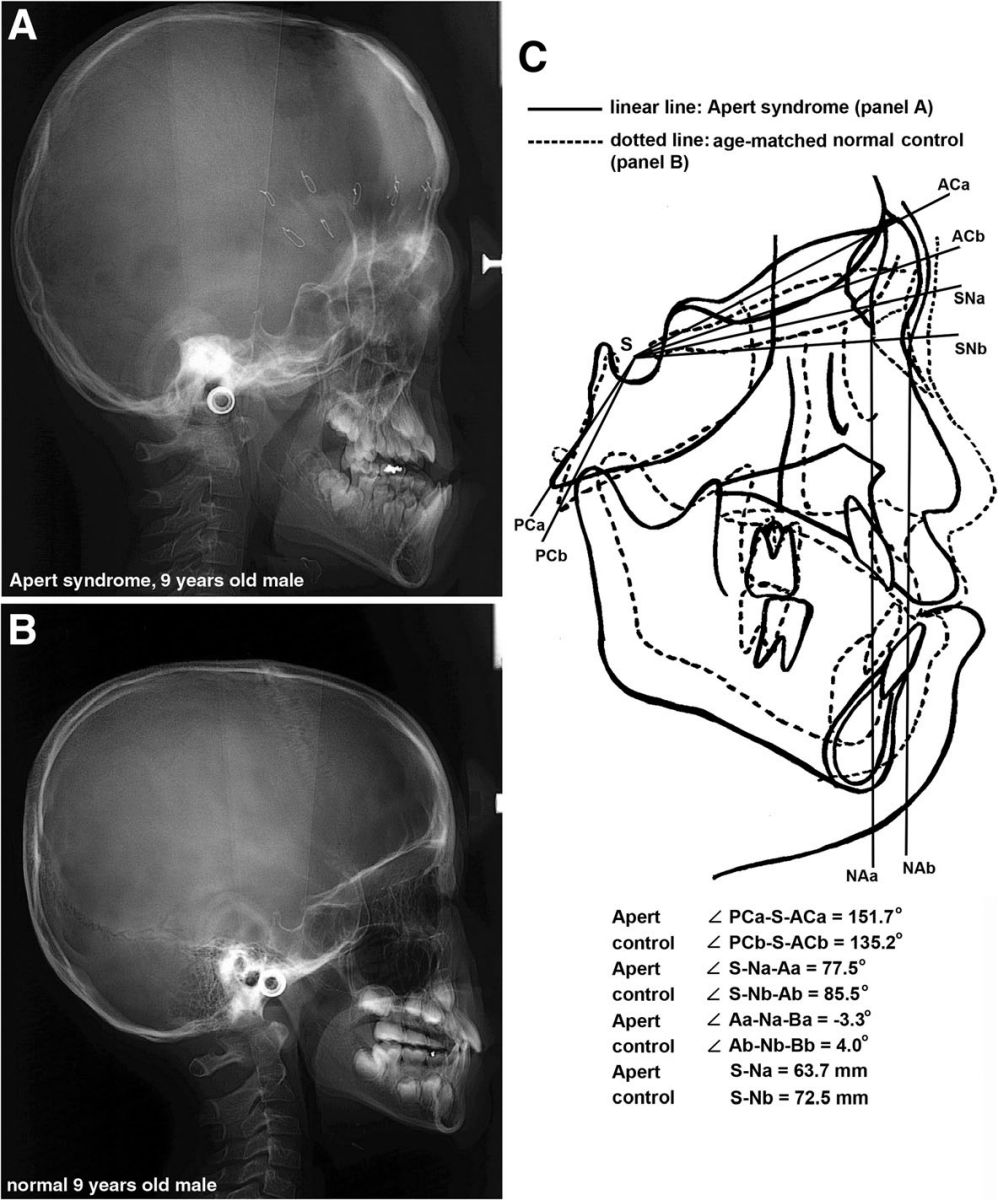
Cephalogram: **a** An Apert syndrome patient, a 9-year-old male, noted with retained suture wires for distraction osteotomy performed at 6 months after birth. **b** A 9-year-old normal male. **c** overlapping cephalogram panels **a** and **b** by adjusting to the SN line. The Apert syndrome patient showed an increased posterior-anterior cranial base angle (angle PC-S-AC), a retruded maxilla, and a counter-clockwise growth of the mandible compared to the control

In the comparison of cephalograms between the Apert syndrome patient and the control, the overlapped tracing images by adjusting the SN line disclosed that the Apert syndrome patient showed decreased length between the sella (S) to nasion (N) (63.7 mm), decreased SNA angle (77.5°), and increased posterior-anterior cranial base angle (angle PC-S-AC, 151.7°) compared to the control (72.5 mm, 85.5°, 135.2°, respectively). The maxillary undergrowth and relative mandibular protrusion was evident, resulting in the reverse angulation of ANB angle (− 3.3°) compared to the ANB angle (4°) of the control. Particularly, compared to the control, the Apert syndrome patient had a smaller nasion, which was also hypoplastic and associated with small nasal septal cartilage retruded markedly (Fig. 4).

**Fig. 5:**
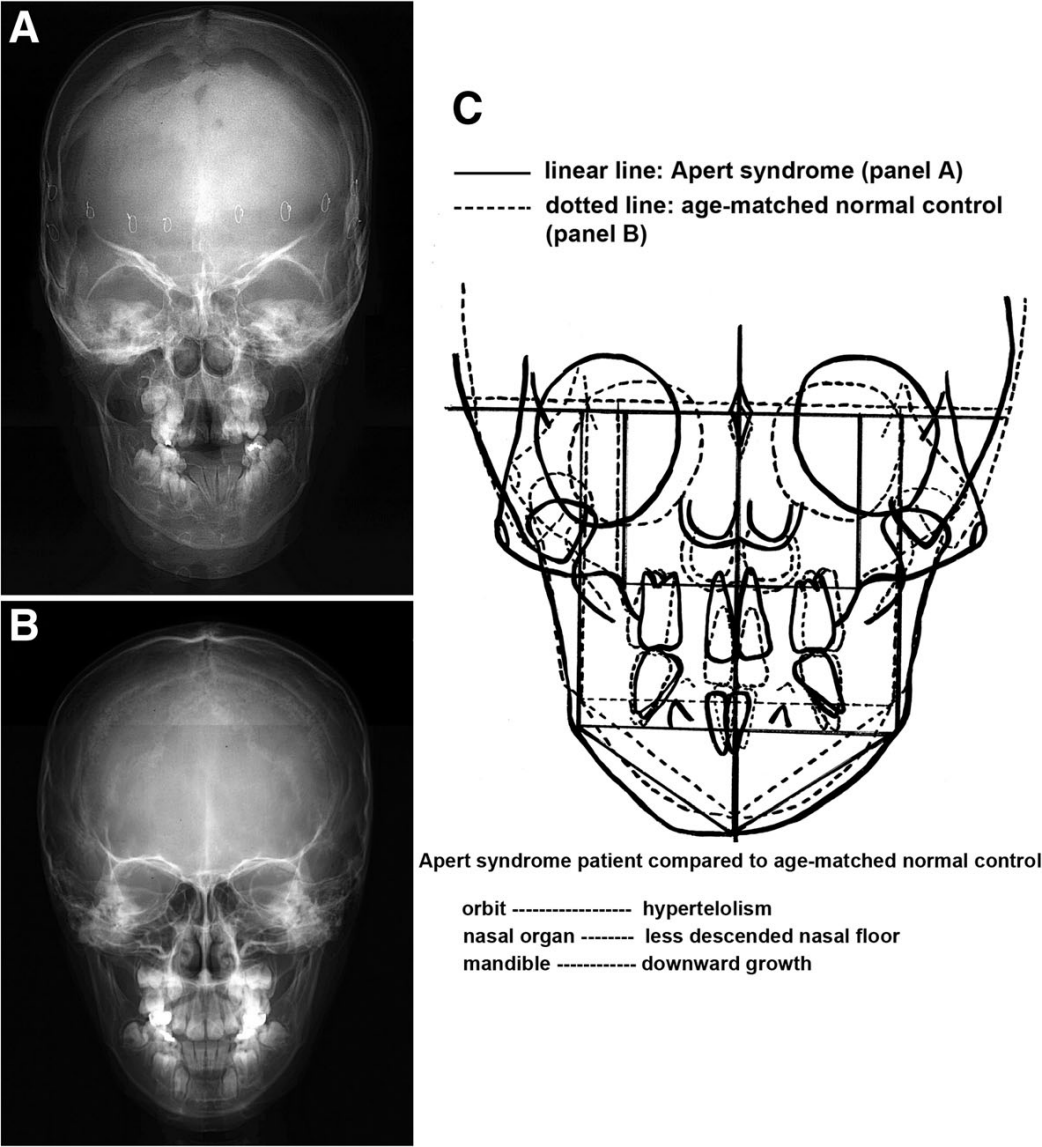
Posterior to anterior (PA) view. **a** An Apert syndrome patient, a 9-year-old male. **b** A 9-year-old normal male. **c** Overlapping panels **a** and **b** by adjusting the line between the bilateral jugular points. The Apert syndrome patient showed orbital hypertelorism, less descended nasal floor, and downward growth of the mandible compared to the control

In the comparison of skull PA views between the Apert syndrome patient and the control, the overlapped tracing images by adjusting the line between bilateral jugular points clearly disclosed that the Apert syndrome patient showed severe orbital hypertelorism with increased length between the centers of bilateral zygomatic bones, less descended nasal floor due to the retarded growth of the nasal organ, and downward growth of the mandible compared to the control (Fig. 5).

**Fig. 6:**
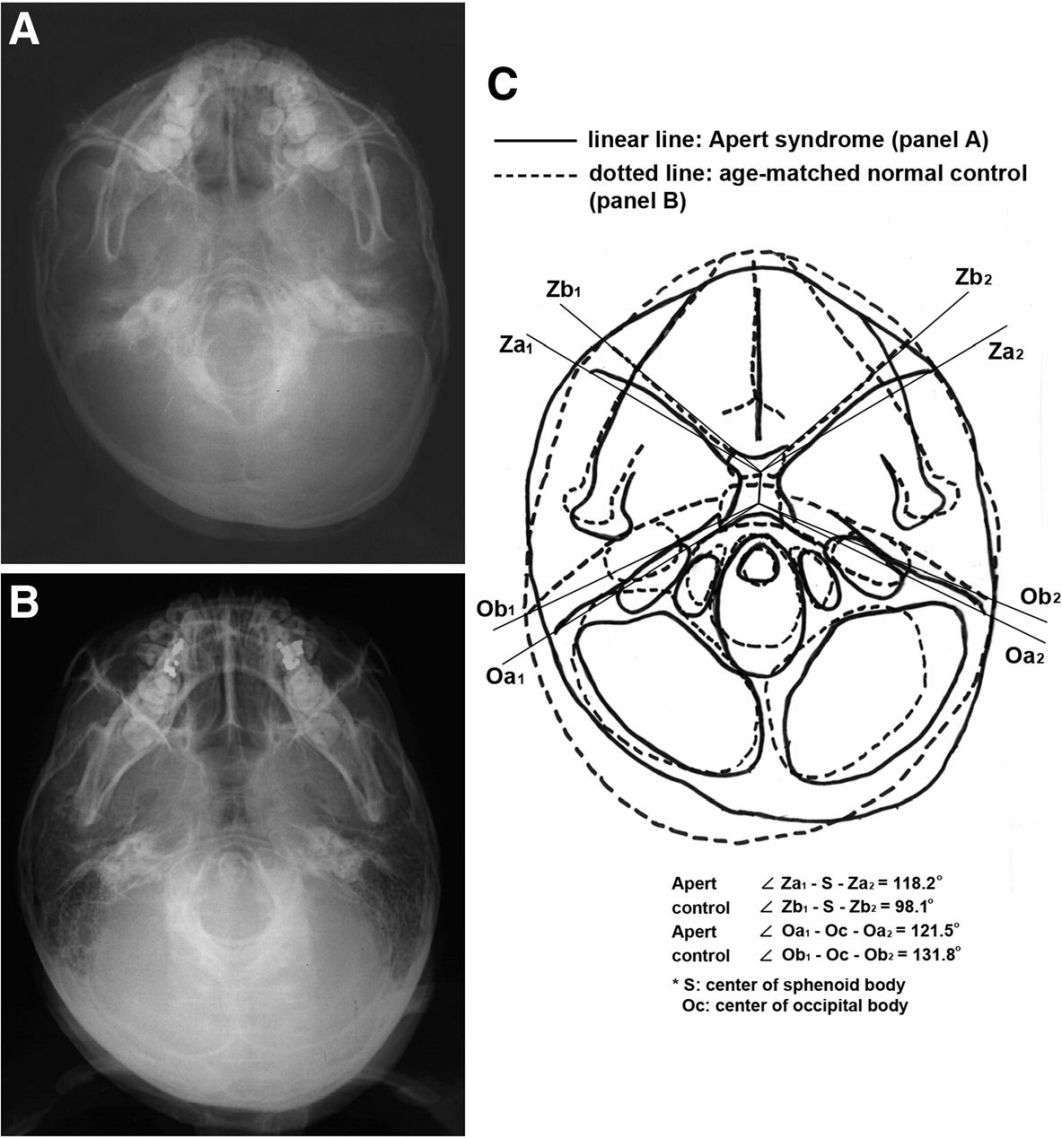
Basal cranial view. **a** An Apert syndrome patient, a 9-year-old male. **b** A 9-year-old normal male. **c** Overlapping panels **a** and **b**. The Apert syndrome patient showed increased zygomatic axis angle of the cranial base (angle Z1-S-Z2) and decreased otic axis angle of the cranial base (angle O1-Oc-O2) compared to the control

In basal cranial view, the Apert syndrome patient showed much bigger zygomatic axis angle of the cranial base (118.2°) and smaller otic axis angle of the cranial base (121.5°) than the control (98.1°, 131.8°, respectively), implicating that the zygomatic axes of the Apert syndrome patient were retruded markedly compared to those of the control, while the otic axes of the Apert syndrome patient were rotated posteriorly compensatory to maintain the middle cranial base volume compared to those of the control (Fig. 6).

**Fig. 7:**
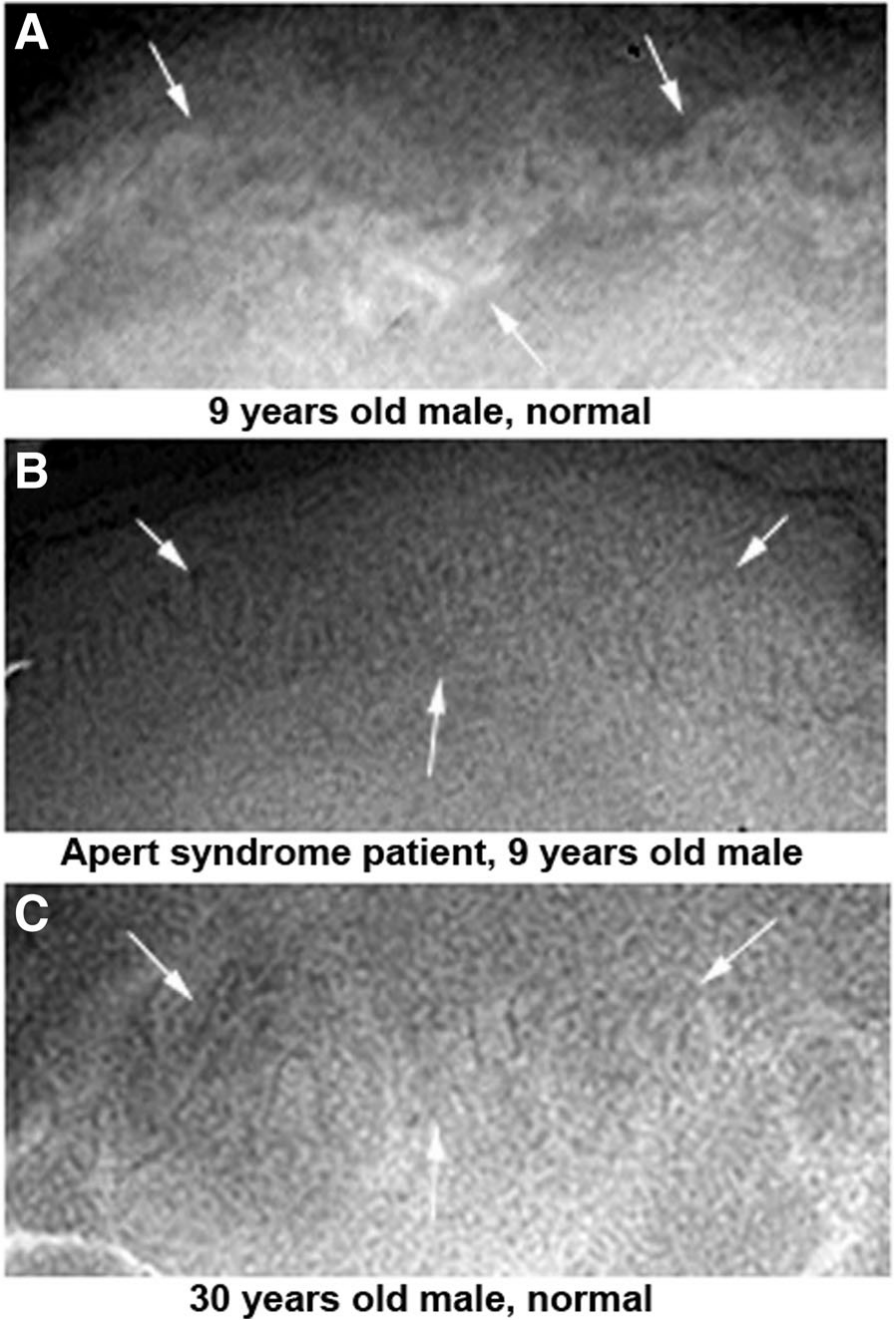
Coronal sutures in PA X-ray views. **a** A 9-year-old normal male with still immature coronal suture (arrows). **b** An Apert syndrome patient, a 9-year-old male, with tightly interdigitated coronal suture (arrows) similar to that of a 30-year-old normal male (**c**)

In the skull PA view, the Apert syndrome patient showed much more interdigitated and mineralized coronal suture than the control. The calvarial suture ossification of the present Apert syndrome patient was similar to that of a 30-year-old normal male selected as a representative one (Fig. 7).

## Discussion

Although the craniofacial structures are complicated and composed of heterogeneous components, their dynamic growth patterns could be well illustrated by different radiological methods. For the precise radiological observation of craniofacial structures, the panthomogram and skull PA view may show the vertical relationships of craniofacial structures with the good images of nasal septal cartilage, and Water’s view may show the naso-maxillary structures parallel to the anterior cranial base plane [14, 15]. Cephalogram may reveal the growth pattern of both jaws and posterior-anterior inclination of the cranial base on the sagittal plane [16]. And the basal cranial view may show the whole cranial base structures composed of spheno-occipital synchondrosis, nasal septum axis, zygomatic axes, and otic axes [17, 18]. However, to get the reliable radiological measurements, every radiogram for craniofacial structures was taken in using the precise methods by the trained expert.

As the Apert syndrome is known as a craniosynostosis originating from an abnormal FGFR2 protein which can produce premature ossification of chondroid and osteoid tissues, the cranial base structures composed of active osteochondroid tissue could be the primary target of Apert syndrome [1–3]. The abnormal growth patterns of the cranial base and midface structures, demonstrated in this study, may be caused by premature osteosynchondrosis of cranial base cartilages, i.e., spheno-occipital cartilage and the associated axial cartilages, which are derived from the same prechordal mesoderm [19].

Particularly, the zygomatic axis angle of the cranial base was much bigger in the Apert syndrome patient, 9-year-old male, than in the control. This fact may imply that in the Apert syndrome the zygomatic axes of the cranial base is premature and ossified before the appropriate anterior rotation of zygomatic axes as usual [19] so that the zygomatic axes of the Apert syndrome patient are hardly able to rotate anteriorly to support the anterior cranial base structures. And subsequently, the anterior cranial base became loosened and hypoplastic together with the undergrowth of nasal organ cartilage [20, 21].

Divergent types of craniofacial synostosis appeared with the diagnosis of scaphocephaly, trigonocephaly, anterior plagiocephaly, occipital plagiocephaly, and non-syndromic multi-suture synostosis besides Crouzon syndrome and Apert syndrome [22]. In Apert syndrome, including cranial deformities and syndactyly (acrocephalosyndactyly), although intracranial hypertension, exophthalmos, and midface hypoplasia were mild, the mandibular distraction, in addition to fronto-orbital distraction, and Le Fort III midface distraction might give good results [23].

The present case of Apert syndrome characteristically exhibited the severe growth retardation of nasal organ cartilage in the observation of all the radiograms, which resulted in the hypoplasia of nasal cavity structures including the nasal septum and nasal space. Subsequently, the aberrant growth of the nasal organ may produce the hypoplastic premaxillo-septal ligament and vomer in the vicinity of the premaxilla and palate and negatively affect the expansile growth of the maxillary arch and palate. The presence of fissure-like groove in the mid-palatal area was coincident with the upward convex and high-positioned palatal plate in the panthomogram and less descended nasal floor in the skull PA view compared to the control. These findings may indicate that the growth of the whole nasal organ including vomer is greatly retarded and became hypoplastic, consequently resulting in the severely retruded maxilla.

Actually, the forward growth of the maxilla cannot be achieved without the tensile force of premaxillo-septal ligament attached between the premaxilla and nasal septum, and the palatal bones have to anchor on the vomer located underneath the nasal septum to get the counter force for the palatal expansion [24–26]. The present Apert syndrome patient showed the severe hypoplasia of the maxilla, accompanied with a less descended nasal floor in the skull PA view and fissure-like groove on the mid-palate area in the intraoral observation.

In Apert syndrome, there is a high incidence of raised intracranial pressure, which can first occur at any age up to 5 years and may recur despite the initial successful treatment of standard frontal-orbital advancement or frontofacial monobloc advancement with pedicled flaps in multiple synostosis, trigonocephalies, and plagiocephalies [27–29]. To ameliorate the brain development, the present Apert syndrome patient received a distraction osteotomy of frontal calvaria at 6 months old, and now at 9 years old, he showed a linear scar zone of new bone formation in the forehead by frontal calvaria expansion. Fortunately, the present patient has no problem in the ear organ so far, but continuous follow-up check should be recommended for possible auditory defects addressed previously [30].

The symptom complex caused by the raised intracranial pressure in Apert syndrome could be a reliable hallmark for the advance of craniosynostosis, which may have been continuously progressed from the developmental stage during the fetal period [31]. However, the fact that the Apert syndrome patient can feel the raised intracranial pressure around 5 years of age may suggest that any surgical or orthodontic treatment should be started at least from 5 years of age.

On the other hand, in the present Apert syndrome patient, a 9-year-old male, the rhomboidal and coronal calvarial suture in the skull PA view was tightly interdigitated and almost calcified compared to the same-age male subject. The suture maturation of the present 9-year-old patient was similar to that of a 30-year-old male observed in this study. Therefore, it was presumed that the craniofacial synostosis was far advanced in the present Apert syndrome patient, so that aberrant growth of the craniofacial structure was supposed to be prematurely ossified to a degree.

## Conclusions

With the radiological observation of the craniofacial structure of the Apert syndrome patient, it was found that the pathogenetic craniosynostosis of Apert syndrome widely occurred not only in the midface structures but also in the basic structures of the cranial base. Especially, the findings that the anterior rotation of zygomatic axes of the cranial base was delayed and still insufficient and the fact that the nasal septum cartilage which functions as an anterior axis of the cranial base was much undergrown may consequently affect the hypoplastic growth of midface structures even after frontal calvaria distraction osteotomy 8 years ago. As the craniofacial structures of Apert syndrome may produce fundamental changes by premature osteosynchondrosis during the early growth stage of postnatal period, it was also suggested that any surgical or orthodontic treatment should be carefully performed to adapt and to modify the abnormal craniofacial structures as early as possible at least from 5 years of age.
